# Inconclusive prospective associations between maternal emotional expressiveness and children’s callous-unemotional traits: a reanalysis and comment on Hu et al. (2026)

**DOI:** 10.3389/fpsyg.2026.1821616

**Published:** 2026-05-18

**Authors:** Kimmo Sorjonen, Bo Melin, Marika Melin

**Affiliations:** Karolinska Institutet (KI), Solna, Sweden

**Keywords:** children’s callous-unemotional traits, cross-lagged panel model (CLPM), juxtaposition of findings, maternal emotional expressiveness, uncertain prospective associations

## Abstract

In a recent study, researchers used cross-lagged panel models (CLPM) and concluded prospective associations between maternal emotional expressiveness and children’s callous-unemotional traits. However, it is well established that associations in the CLPM may be uncertain. Here, we fitted alternative models to data simulated to resemble data in the original study. We report discrepant increasing, decreasing, and null associations depending on the analyzed model and meta-analytic aggregations of these discrepant associations did not differ significantly from zero. Hence, we propose that findings in the original study may have been uncertain and conclusions premature. It is important for researchers to bear in mind that associations in observational (i.e., non-experimental) data, including cross-lagged effects in the CLPM, may be uncertain in order not to overinterpret findings. We recommend researchers to fit alternative models to data and to base conclusions on a juxtaposition of findings.

## Introduction

### The cross-lagged panel model (CLPM)

**Figure 1:**
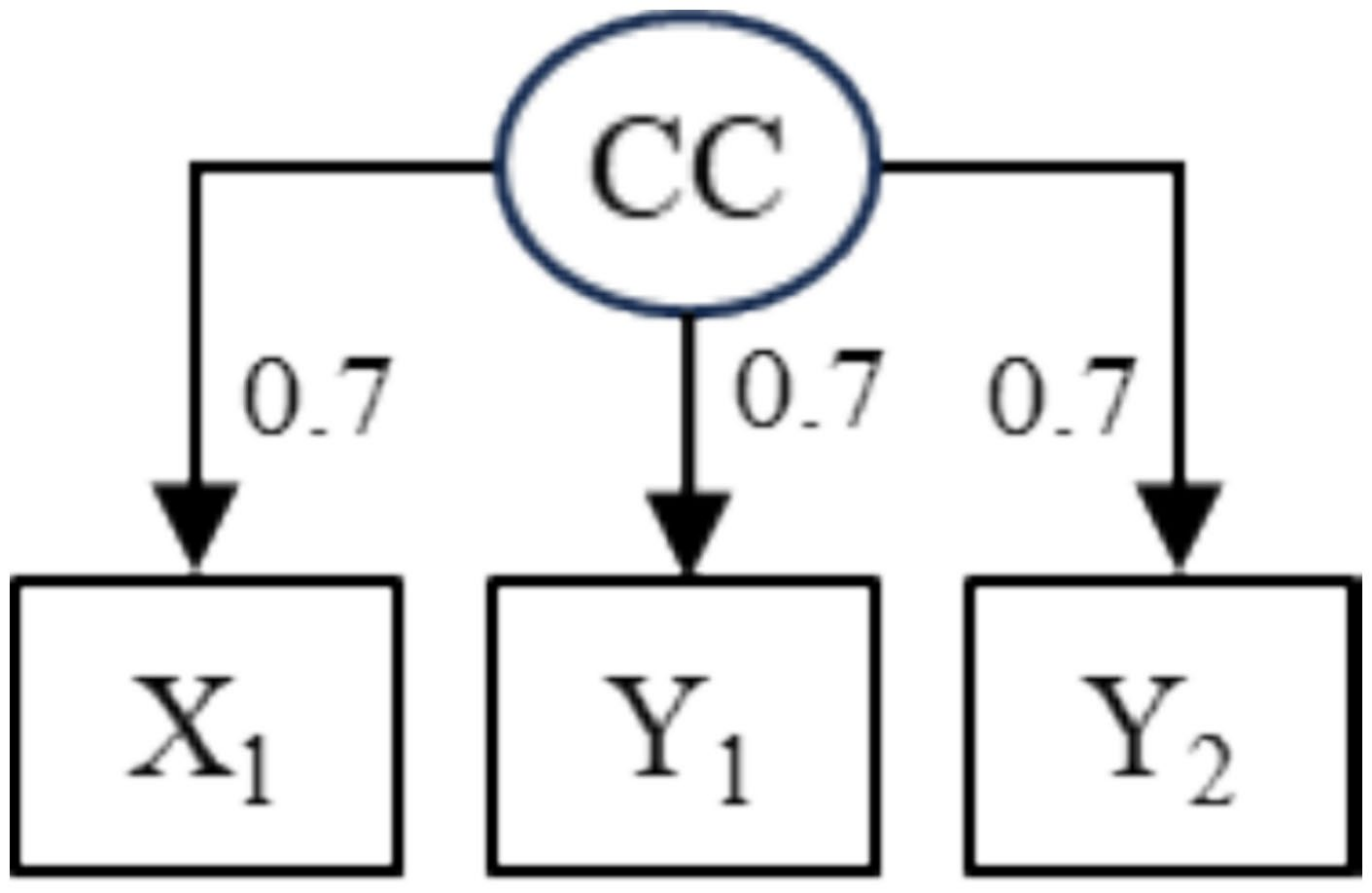
Model that would generate data with an uncertain cross-lagged effect *β* = 0.33 of *X*_1_ on *Y*_2_ when adjusting for *Y*_1_. CC, common cause.

In cross-lagged panel models (CLPM), subsequent scores on two (or more) variables are regressed on initial scores on the same variables. Cross-lagged effects of *X*_1_ on *Y*_2_ when adjusting for *Y*_1_ are often assumed, at least in psychological research, to allow stronger inference compared to cross-sectional and cross-lagged correlations. For example, Hu et al. (2026) analyzed longitudinal data (*N* = 561) on maternal emotional expressiveness and children’s callous-unemotional traits (both variables rated by the mothers) across three waves of measurement with the CLPM and concluded a dynamic interplay between these constructs. However, it is well established that cross-lagged effects may be uncertain (e.g., Campbell and Kenny, 1999). For example, would data be generated as in Figure 1, the cross-lagged effect of *X*_1_ on *Y*_2_ when adjusting for *Y*_1_ would be *β* = 0.33 although *X*_1_ has no direct effect on *Y*_2_, meaning that the effect would be uncertain.

We (Sorjonen et al., 2025) have proposed that cross-lagged effects may be scrutinized by estimating the effect of *X*_1_ on the *Y*_2_ – *Y*_1_ difference score when adjusting for *Y*_1_ (Equation 1), when adjusting for *Y*_2_ (Equation 2), and when not adjusting for *Y*_1_ or *Y*_2_ (Equation 3). Then, these three effects (i.e., coefficients *a*_1_, *b*_1_, and *c*_1_ in Equations 1–3, respectively) may be meta-analytically pooled and conclusions based on the pooled effect. When adjusting for *Y*_1_, the effect of *X*_1_ on the *Y*_2_ – *Y*_1_ difference (coefficient *a*_1_ in Equation 1) is identical to the traditional cross-lagged effect of *X*_1_ on *Y*_2_. Estimating the effect of *X*_1_ on the *Y*_2_ – *Y*_1_ difference when adjusting for *Y*_2_ (coefficient *b*_1_ in Equation 2) is in line with Campbell and Kenny’s (1999) recommendation to use time-reversed analyses to identify statistical artifacts. Our recommendation to fit alternative reasonable models to data and to base conclusions on a juxtaposition of findings is in line with multiverse methodology (Steegen et al., 2016, see also Olsson-Collentine et al., 2023).


$$Y_{2}-Y_{1}=a_{0}+a_{1}X_{1}+a_{2}Y_{1}+e$$ (1)

$$Y_{2}-Y_{1}=b_{0}+b_{1}X_{1}+b_{2}Y_{2}+e$$ (2)

$$Y_{2}-Y_{1}=c_{0}+c_{1}X_{1}+e$$ (3)

## Our reanalyses

We simulated data to resemble the data used by Hu et al. (2026), with the same sample size, three waves of measurements, and the same correlations between variables (these correlations were reported by Hu et al. (2026)). We used simulated data as the empirical data was not available to us. The corresponding author of the study by Hu et al. (2026) did not respond to our request for the data. Here, it is important to note that standardized regression effects are functions of correlations and will be the same in data with the same correlations, irrespective if the data is empirical or simulated. Given standardized (*M* = 0, SD = 1) variables *X*_1_, *Y*_1_, and *Y*_2_, effects a_1_, b_1_, and c_1_ in Equations 1–3 are given by Equations 4–6 (which are based on standard equations for adjusted regression effects, see Cohen et al., 2003), respectively. This means that the effects are fully defined by correlations between the variables (*r*_*x*1,*y*1_, *r*_*x*1,*y*2_, and *r*_*y*1,*y*2_, respectively) irrespective of, for example, distributions of the variables. Hence, the findings reported below approximates what the findings would have been if estimated in the empirical data used by Hu et al. (2026)


$$a_{1}=\frac{r_{x1,y2}-r_{x1,y1}r_{y1,y2}}{1-r_{x1,y1}^{2}}$$ (4)

$$b_{1}=\frac{r_{x1,y2}r_{y1,y2}-r_{x1,y1}}{1-r_{x1,y2}^{2}}$$ (5)

$$c_{1}=r_{x1,y2}-r_{x1,y2}$$ (6)

We estimated effects (coefficients *a*_1_, *b*_1_, and *c*_1_ in Equations 1–3, respectively) of maternal positive and negative emotional expressiveness on subsequent change in children’s callous-unemotional traits, and vice versa, separately for the timeframes time 1 to time 2 and time 2 to time 3 (i.e., six effects in total) and pooled these effects with a random-effects meta-analytic model. For the analyses and simulations we used R 4.4.3 statistical software (R Core Team, 2026) employing the MASS (Venables and Ripley, 2002), metafor (Viechtbauer, 2010), and lavaan (Rosseel, 2012) packages. The analytic script, which also generates the simulated data, is available at the Open Science Framework at https://osf.io/k9b65/.

**Figure 2:**
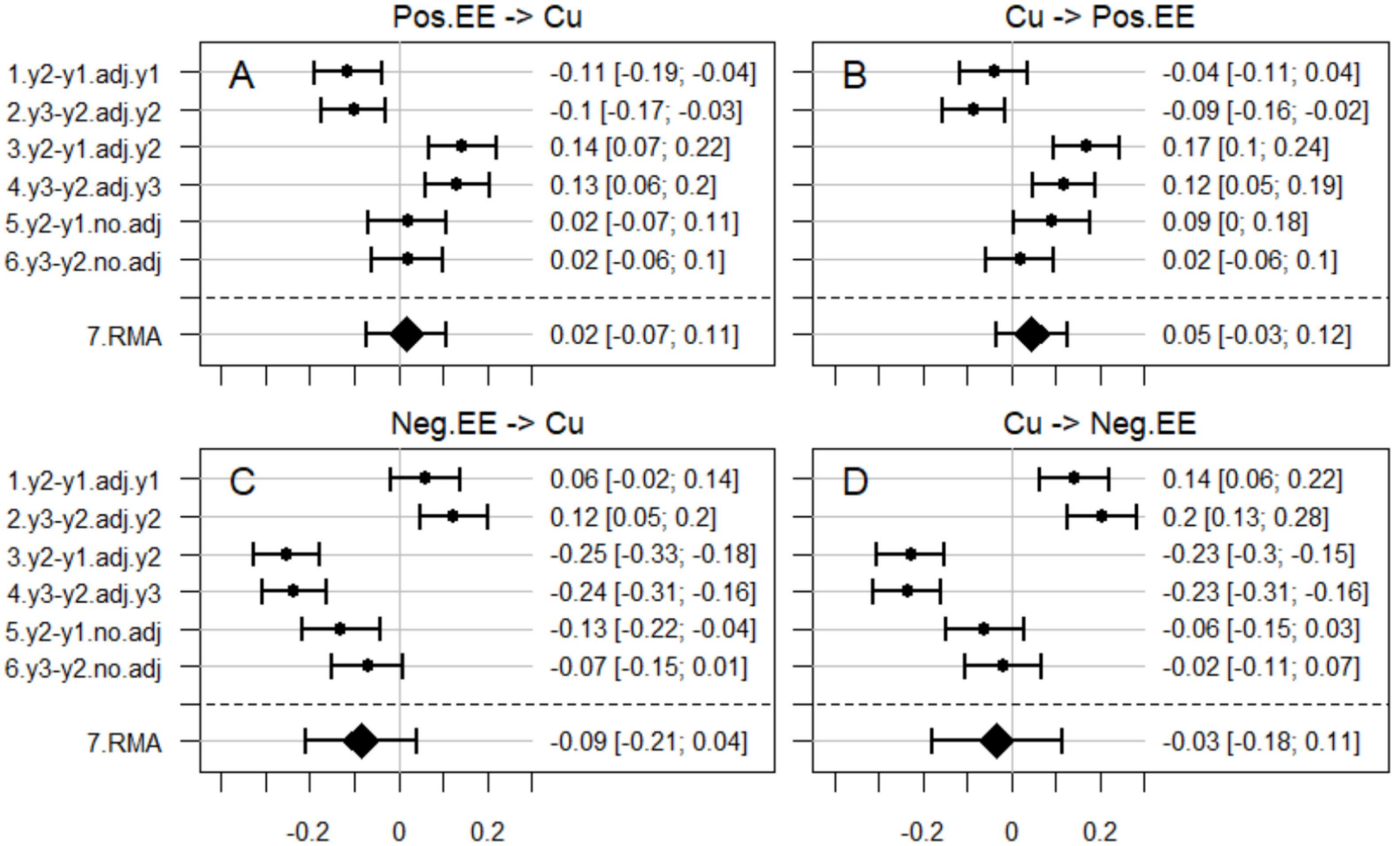
Effects of maternal positive emotional expressiveness on subsequent change in children’s callous-unemotional traits **(A)** and vice versa **(B)**, as well as effects of maternal negative emotional expressiveness on subsequent change in children’s callous-unemotional traits **(C)** and vice versa **(D)**. Note: effects with numbers 1 and 2 were adjusted for an initial score on the outcome (corresponding to coefficient *a*_1_ in Equation 1), effects with numbers 3 and 4 for a subsequent score on the outcome (corresponding to coefficient *b*_1_ in Equation 2), and effects with numbers 5 and 6 neither for an initial nor a subsequent score on the outcome (corresponding to coefficient *c*_1_ in Equation 3). Effects with number 7 are random models meta-analytic (RMA) poolings of the six effects.

In line with findings by Hu et al. (2026), maternal positive emotional expressiveness had a negative and negative emotional expressiveness a positive association with subsequent change in children’s callous-unemotional traits, and vice versa, when adjusting for an initial score on the outcome (effects with numbers 1 and 2 in Figure 2, corresponding to coefficient *a*_1_ in Equation 1). However, when adjusting for a subsequent instead for an initial score on the outcome, these effects changed signs (effects with numbers 3 and 4 in Figure 2, corresponding to coefficient *b*_1_ in Equation 2). Moreover, when not adjusting for an initial or a subsequent score on the outcome, the effect of initial maternal emotional expressiveness on subsequent change in children’s callous-unemotional traits, and vice versa, tended to be weak and in most cases statistically non-significant (effects with numbers 5 and 6 in Figure 2, corresponding to coefficient *c*_1_ in Equation 3). Meta-analytic poolings of these discrepant effects were statistically non-significant (effects with number 7 in Figure 2). Detailed descriptions of the effects in Figure 2 are presented in Table 1.

**Table 1:** Detailed description of the effects in Figure 2.

Panel	Effect	Description
A	1	The effect of MPEE at time 1 on the CU at time 2—CU at time 1 difference score when adjusting for CU at time 1
A	2	The effect of MPEE at time 2 on the CU at time 3—CU at time 2 difference score when adjusting for CU at time 2
A	3	The effect of MPEE at time 1 on the CU at time 2—CU at time 1 difference score when adjusting for CU at time 2
A	4	The effect of MPEE at time 2 on the CU at time 3—CU at time 2 difference score when adjusting for CU at time 3
A	5	The unadjusted effect of MPEE at time 1 on the CU at time 2—CU at time 1 difference score
A	6	The unadjusted effect of MPEE at time 2 on the CU at time 3—CU at time 2 difference score
A	7	Meta-analytic pooling of effects 1–6 in panel A
B	1	The effect of CU at time 1 on the MPEE at time 2—MPEE at time 1 difference score when adjusting for MPEE at time 1
B	2	The effect of CU at time 2 on the MPEE at time 3—MPEE at time 2 difference score when adjusting for MPEE at time 2
B	3	The effect of CU at time 1 on the MPEE at time 2—MPEE at time 1 difference score when adjusting for MPEE at time 2
B	4	The effect of CU at time 2 on the MPEE at time 3—MPEE at time 2 difference score when adjusting for MPEE at time 3
B	5	The unadjusted effect of CU at time 1 on the MPEE at time 2—MPEE at time 1 difference score
B	6	The unadjusted effect of CU at time 2 on the MPEE at time 3—MPEE at time 2 difference score
B	7	Meta-analytic pooling of effects 1–6 in panel B
C	1	The effect of MNEE at time 1 on the CU at time 2—CU at time 1 difference score when adjusting for CU at time 1
C	2	The effect of MNEE at time 2 on the CU at time 3—CU at time 2 difference score when adjusting for CU at time 2
C	3	The effect of MNEE at time 1 on the CU at time 2—CU at time 1 difference score when adjusting for CU at time 2
C	4	The effect of MNEE at time 2 on the CU at time 3—CU at time 2 difference score when adjusting for CU at time 3
C	5	The unadjusted effect of MNEE at time 1 on the CU at time 2—CU at time 1 difference score
C	6	The unadjusted effect of MNEE at time 2 on the CU at time 3—CU at time 2 difference score
C	7	Meta-analytic pooling of effects 1–6 in panel C
D	1	The effect of CU at time 1 on the MNEE at time 2—MNEE at time 1 difference score when adjusting for MNEE at time 1
D	2	The effect of CU at time 2 on the MNEE at time 3—MNEE at time 2 difference score when adjusting for MNEE at time 2
D	3	The effect of CU at time 1 on the MNEE at time 2—MNEE at time 1 difference score when adjusting for MNEE at time 2
D	4	The effect of CU at time 2 on the MNEE at time 3—MNEE at time 2 difference score when adjusting for MNEE at time 3
D	5	The unadjusted effect of CU at time 1 on the MNEE at time 2—MNEE at time 1 difference score
D	6	The unadjusted effect of CU at time 2 on the MNEE at time 3—MNEE at time 2 difference score
D	7	Meta-analytic pooling of effects 1–6 in panel D

MPEE, maternal positive emotional expressiveness; MNEE, maternal negative emotional expressiveness; CU, children’s callous-unemotional traits.

## The model of spurious longitudinal associations (MoSLA)

**Figure 3:**
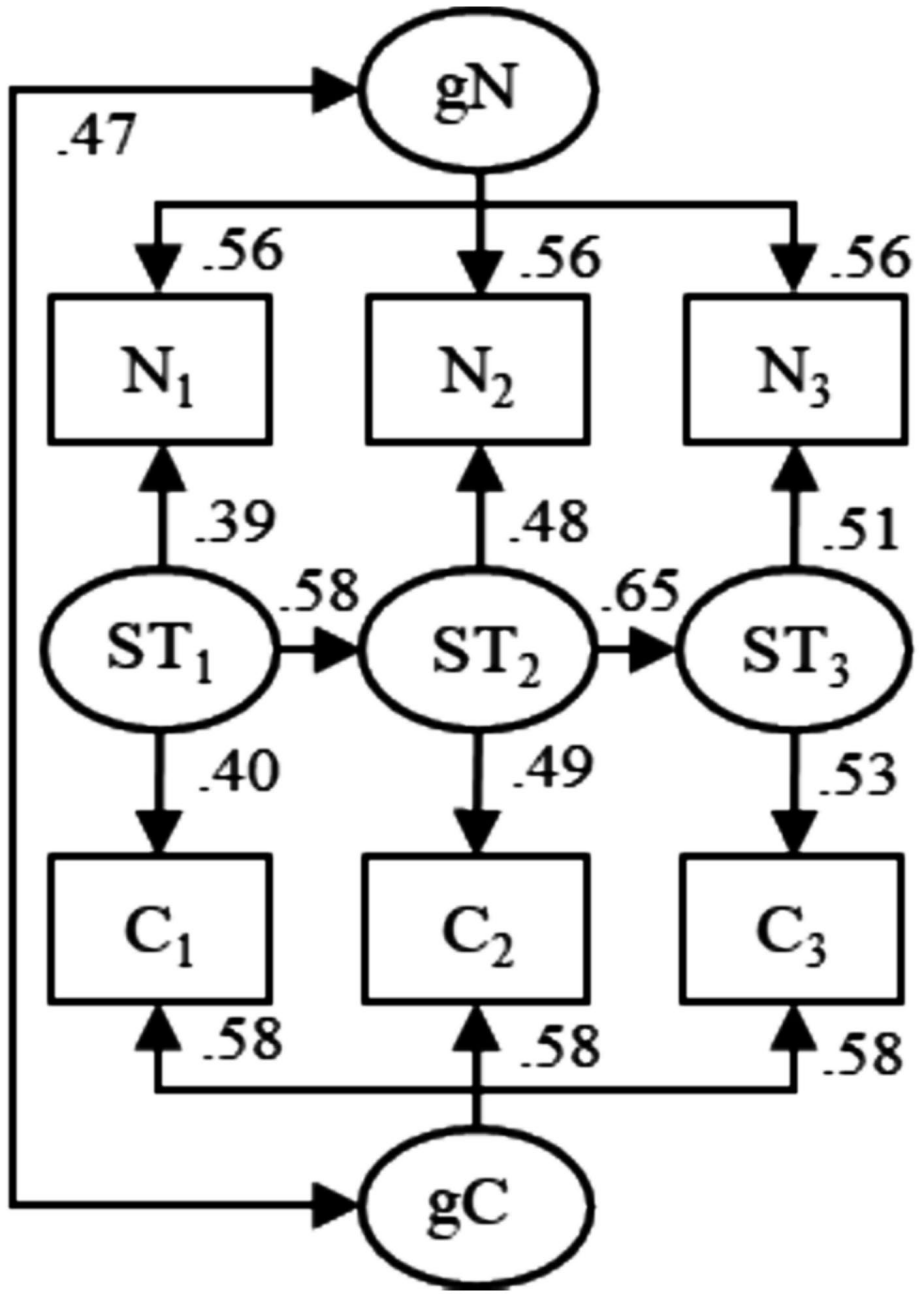
The model of spurious longitudinal associations (MoSLA) fitted to simulated data (*N* = 561) on maternal negative emotional expressiveness (N) and children’s callous-unemotional traits (C) across three waves of measurement (the subscripts 1, 2, and 3, respectively). Observed scores on the two constructs were regressed on general trait-like levels of negative emotional expressiveness (gN) and callous traits (gC), respectively, as well as occasion-specific (but autocorrelated) state-factors (ST), standardized estimates. All parameters were highly statistically significant (*p* < 0.001). Rectangles indicate empirically observed scores while circles indicate implied latent variables. Single-headed arrows indicate unidirectional regression effect while double-headed arrows indicate correlations. The model had excellent fit (*χ*^2^ = 10.4, DF = 15, *p* = 0.791, CFI = 1.000, TLI = 1.006, RMSEA = 0.000 [90% CI: 0.000; 0.027]).

Instead of assuming genuine prospective effects between maternal emotional expressiveness and children’s callous-unemotional traits, not supported by the findings above, we propose that the data analyzed by Hu et al. (2026), and simulated and reanalyzed by us, may have been generated by the model of spurious longitudinal associations (MoSLA, Sorjonen and Melin, 2025a,b) (Figure 3). The MoSLA assumes that longitudinal scores on two constructs are affected by stable trait-like levels of the constructs as well as by common occasion-specific (but autocorrelated) state-factors. However, the MoSLA does not assume direct effects between the measured scores, meaning that such effects, if identified by statistical analyses (e.g., the CLPM), would be uncertain.

When fitted to the present simulated data on maternal negative emotional expressiveness and children’s callous-unemotional traits, the model had excellent fit (Figure 3). This suggests that the data analyzed by Hu et al. (2026), and simulated and reanalyzed by us, may have been generated without any direct effects between the constructs and that the cross-lagged effects reported by Hu et al. (2026) may have been uncertain. Here, the common state-factors, affecting both self-rated emotional expressiveness and ratings of children’s callous-unemotional traits, might have been factors such as mood, employment situation, conflict with partner, etc. Hu et al. (2026) noted that reliance solely on mother-reported data introduced potential social desirability and common method bias, which may also have contributed to uncertain associations.

## Limitations (and response)

The present reanalyses do not prove the null hypothesis of no genuine (i.e., non-spurious) prospective associations between maternal emotional expressiveness and children’s callous-unemotional traits. The more limited conclusion to be drawn is that the data analyzed by Hu et al. (2026), and simulated and reanalyzed by us, do not prove that the null hypothesis is false. On a similar note, the excellent fit of the MoSLA (Figure 3) does not prove that the MoSLA was the true data-generating model, as other well-fitting models are possible. However, the excellent fit of the MoSLA indicates that data may have been generated without direct effects between the longitudinal scores on maternal emotional expressiveness and children’s callous-unemotional traits. One advantage of the MoSLA compared with models assuming genuine prospective associations between maternal emotional expressiveness and children’s callous-unemotional traits, e.g., the CLPMs reported by Hu et al. (2026), is that it is not contradicted by the discrepant effects reported in the present study (see Figure 2). However, we do not claim that the MoSLA is more plausible than alternative models. Without empirical comparison, the MoSLA is better framed as illustrative.

Here, we limited our analyses to multiverse regression analyses (with meta-analytic pooling) of the effect of initial maternal emotional expressiveness on subsequent change in children’s callous-unemotional traits and vice versa (Equations 1–3) as well as the MoSLA (Figure 3). However, it should be noted that Hu et al. (2026) acknowledged limitations of the CLPM and they analyzed data also with parallel process latent growth curve models (LGCM), which indicated co-development of maternal negative emotional expressiveness and children’s callous-unemotional traits across the waves of measurement. Moreover, Hu et al. (2026) mentioned that future research would benefit from employing the random-intercept cross-lagged panel model (RI-CLPM, Hamaker et al., 2015; Mulder and Hamaker, 2021). However, it is important to note that when fitted to observational (i.e., non-experimental) data, effects in both the LGCM and the RI-CLPM may be uncertain. For example, the RI-CLPM cannot adjust for time-varying confounders (Murayama and Gfrörer, 2024; Rohrer and Murayama, 2023). This means that the RI-CLPM is susceptible to similar uncertain findings as the traditional CLPM (Sorjonen and Melin, 2025b). Hence, (potentially) good fit of and statistically significant effects in the LGCM and the RI-CLPM would not nullify the discrepant effects (Figure 2) and the good fit of the MoSLA (Figure 3) reported here. This means that the association in the LGCM reported by Hu et al. (2026), and potential cross-lagged effects in the RI-CLPM, are (would be) compatible with our conclusion that the findings by Hu et al. (2026) may have been uncertain and that their conclusion of dynamic interplay between maternal emotional expressiveness and children’s callous-unemotional traits may have been premature.

As mentioned above, the present study was limited to multiverse regression analyses and the MoSLA. For improved causal inference in developmental psychology, researchers have also recommended using, for example, fixed effects regression, propensity score methods, directed acyclic graphs (DAGs), instrumental variable (IV) estimations, and natural experiments (Foster, 2010; Miller et al., 2016). However, the objective of the present study was to scrutinize Hu et al.’s (2026) findings by analyses with the CLPM and to evaluate if their data unequivocally supported their conclusions. Hence, using such recommended methods was beyond the scope of the present study.

It should be noted that we do not challenge Hu et al.’s (2026) findings *per se*, e.g., the size of their reported cross-lagged regression effects. Our challenge is limited to their conclusion that their observational (i.e., non-experimental) data supported concluding a dynamic interplay between maternal emotional expressiveness and children’s callous-unemotional traits. As shown in the present study, the data could be used to support diametrically different conclusions (Figure 2) and could have been generated without direct effects between the measured scores (Figure 3). The present analyses demonstrate that multiple interpretations are possible, not that the original interpretation by Hu et al. (2026) is unsupported. Moreover, we should clarify that our critique targets stronger causal interpretations rather than more modest claims of dynamic interplay and selective associations.

## Summary and concluding remarks

Here, we showed that data analyzed by Hu et al. (2026) could be claimed to support both increasing, decreasing, and null prospective associations between maternal emotional expressiveness and children’s callous-unemotional traits. Meta-analytic poolings of these discrepant associations did not differ significantly from zero. Moreover, good fit of the MoSLA indicated that data may have been generated without direct effects between longitudinal scores on emotional expressiveness and callous-unemotional traits. Hence, we propose that the findings by Hu et al. (2026) may have been uncertain and that their conclusion of prospective associations may be challenged. The associations reported by Hu et al. (2026) may, for example, have been due to shared method variance due to the single-informant design.

It is important for researchers to bear in mind that correlations in observational (i.e., non-experimental) data may be uncertain. This is true also for superficially more advanced correlations such as adjusted cross-lagged regression effects. We recommend researchers, e.g., in developmental psychology, to analyze data with alternative models and to juxtapose findings, e.g., through meta-analytic pooling. If findings from alternative models point in the same direction, and the meta-analytic association differs significantly from zero, conclusions may be drawn with increased confidence. If, on the other hand and as in the present case, findings from alternative models diverge, confident conclusions should probably be suspended. Our analytic script, available at https://osf.io/k9b65/, may be modified to be used with other datasets.

## Data Availability

Publicly available datasets were analyzed in this study. This data can be found here: The analytic script, which also generates the simulated data, is available at the Open Science Framework at https://osf.io/k9b65/.
